# Particulate substrate retention in plug-flow and fully-mixed conditions during operation of aerobic granular sludge systems

**DOI:** 10.1016/j.wroa.2020.100075

**Published:** 2020-10-28

**Authors:** M. Layer, K. Bock, F. Ranzinger, H. Horn, E. Morgenroth, N. Derlon

**Affiliations:** a Eawag, Swiss Federal Institute of Aquatic Science and Technology, Überlandstrasse 133, 8600, Dübendorf, Switzerland; b ETH Zürich, Institute of Environmental Engineering, 8093, Zürich, Switzerland; c Engler-Bunte-Institut, Karlsruhe Institute of Technology, 76131, Karlsruhe, Germany

**Keywords:** Aerobic granular sludge, Municipal wastewater, Particulate substrate, Plug-flow feeding, Attachment, Flocs

## Abstract

Particulate substrate (X_B_) is the major organic substrate fraction in most municipal wastewaters. However, the impact of X_B_ on aerobic granular sludge (AGS) systems is not fully understood. This study evaluated the physical retention of X_B_ in AGS sequencing batch reactor (SBR) during anaerobic plug-flow and then aerobic fully-mixed conditions. The influence of different sludge types and operational variables on the extent and mechanisms of X_B_ retention in AGS SBR were evaluated. X_B_ mass-balancing and magnetic resonance imaging (MRI) were applied. During the anaerobic plug-flow feeding, most X_B_ was retained in the first few cm of the settled sludge bed within the interstitial voids, where X_B_ settled and accumulated ultimately resulting in the formation of a filter-cake. Sedimentation and surface filtration were thus the dominant X_B_ retention mechanisms during plug-flow conditions, indicating that contact and attachment of X_B_ to the biomass was limited. X_B_ retention was variable and influenced by the X_B_ influent concentration, sludge bed composition and upflow feeding velocity (v_ww_). X_B_ retention increased with larger X_B_ influent concentrations and lower v_ww_, which demonstrated the importance of sedimentation on X_B_ retention during plug-flow conditions. Hence, large fractions of influent X_B_ likely re-suspended during aerobic fully-mixed conditions, where X_B_ then preferentially and rapidly attached to the flocs. During fully-mixed conditions, increasing floc fractions, longer mixing times and larger X_B_ concentrations increased X_B_ retention. Elevated X_B_ retention was observed after short mixing times < 60 min when flocs were present, and the contribution of flocs towards X_B_ retention was even more pronounced for short mixing times < 5 min. Overall, our results suggest that flocs occupy an environmental niche that results from the availability of X_B_ during aerobic fully-mixed conditions of AGS SBR. Therefore, a complete wash-out of flocs is not desirable in AGS systems treating municipal wastewater.

## Highlights

• Fate of particulate substrate (XB) during AGS SBR operation was studied. .
• During plug-flow feeding, XB was retained within the granule bed but didn’t attach. .
• XB preferentially attached to flocs during fully-mixed conditions. .
• Flocs were important for AGS systems in order to remove XB. .

## Graphical abstract

**Figure:**
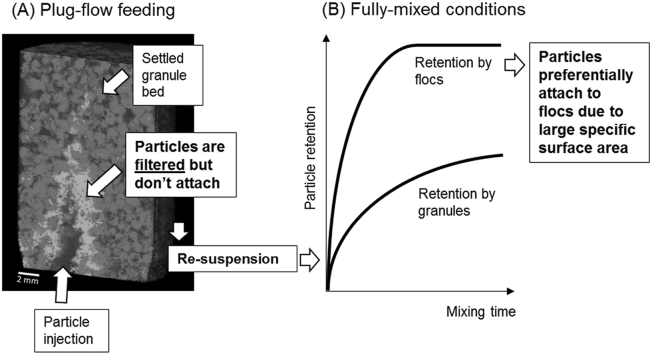

## 1 Introduction

Our understanding of the effect of particulate organic substrate (X_B_) on the formation, operation and overall process performance of aerobic granular sludge (AGS) remains limited, despite many full-scale installations (Derlon et al., 2016; Ali et al., 2019). Prior studies suggested that X_B_ might have several different effects on the behaviour and performance of AGS systems: (1) floc formation and reduced settleability (Wagner et al., 2015b; Derlon et al., 2016; Layer et al., 2019), (2) longer start-up duration (Wagner et al., 2015a; Layer et al., 2019), (3) reduced nutrient removal capability (De Kreuk et al., 2010; Jabari et al., 2016; Guimarães et al., 2018), and (4) deterioration of effluent quality due to an increased effluent solids concentration (Rocktäschel et al., 2015; Van Dijk et al., 2018). However, the link between these observations and the presence of X_B_ in the influent wastewater (WW) is not well understood yet. Research on the overall impact and utilisation pathways of X_B_ on AGS systems is therefore necessary.

X_B_ represents a major fraction of the organic substrate present in municipal WW (typically > 50%) (Metcalf and Eddy, 2014). Hydrolysis of X_B_ is required prior to its utilisation, which often is considered the rate limiting step in biological WW treatment (Morgenroth et al., 2002). In AGS systems, an anaerobic feeding phase of 1–2 h duration - most of the time as plug-flow - is typically applied (Pronk et al., 2015b). However, such period of plug-flow feeding is likely too short to allow for full hydrolysis of X_B_ (Jabari et al., 2016; Wagner et al., 2015b). Therefore, it is suspected that some X_B_ could “leak” into aerobic conditions in AGS operation. The presence of organic substrate in aerobic conditions favours the growth of finger-type granules (De Kreuk et al., 2010; Pronk et al., 2015a) or can even result in process breakdown due to granule breakage (Sturm et al., 2004). However, finger-type granules are rarely observed in AGS systems treating municipal WW (Pronk et al., 2015b; Derlon et al., 2016). Rather, a noticeable growth of flocs is actually observed, so that flocs represent a substantial fraction of 10–20% of the AGS formed during treatment of municipal WW (Wagner et al., 2015a; Derlon et al., 2016; Layer et al., 2019; Pronk et al., 2015b). The presence of flocs in AGS is now acknowledged in full-scale installations (Van Dijk et al., 2018), despite their origin is not well understood. AGS is therefore step-by-step seen as hybrid system, where biofilm (granules) and suspended biomass (flocs) coexist (Layer et al., 2019). Understanding the connection between influent X_B_ and the presence and role of flocs is therefore required.

**Fig. 1:**
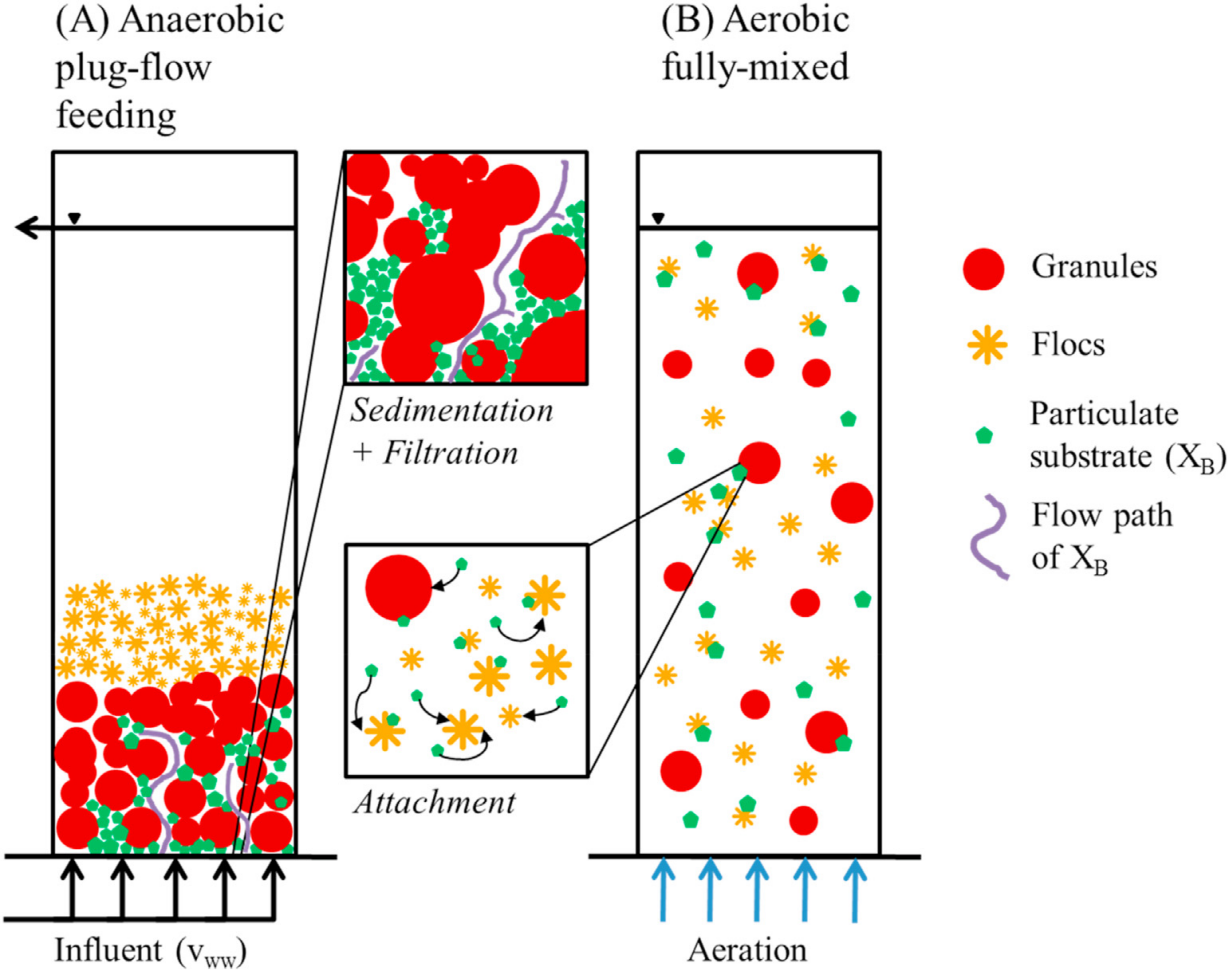
Hypothesized fate of X_B_ during (A) anaerobic plug-flow and (B) aerobic fully mixed conditions. Case (A) illustrates the hypothesized transport and retention pathways of X_B_ during anaerobic plug-flow feeding (retention through sedimentation and surface filtration without much contact or attachment to biomass). Under fully mixed conditions (B) it is hypothesized that X_B_ preferentially attaches to flocs rather than granules.

X_B_ degradation is a three step process: (1) physical contact to biomass (physical X_B_ retention), (2) initiation of enzymatic hydrolysis after contact to biomass, and (3) further utilisation of hydrolysis products as readily biodegradable substrate (S_B_) in anaerobic (fermentation, storage), anoxic (denitrification) or aerobic (direct oxidation, storage) processes. The present study focuses specifically on physical retention of X_B_. Several aspects might hamper physical retention of X_B_ in AGS in comparison to conventional activated sludge systems: (1) distinct hydraulic conditions during anaerobic plug-flow feeding followed by aerobic fully-mixed conditions in SBR operation and (2) the presence of both biofilms (granules) and suspended biomass (flocs) in AGS systems treating municipal WW. Fig. 1 illustrates the possible pathways of X_B_ retention during anaerobic plug-flow feeding (Fig. 1A) and aerobic fully mixed conditions (Fig. 1B). Plug-flow feeding from the bottom of the reactor into the settled AGS bed could limit attachment through restricted contact between influent X_B_ and biomass. If X_B_ then re-suspends during fully mixed conditions, it is hypothesized that flocs would have a competitive advantage in capturing X_B_, due to their much increased adsorption capacity (Andreadakis, 1993) (Fig. 1B). Understanding when, *i.e.*, during plug-flow or fully-mixed conditions, and where, *i.e.*, by flocs or at the granules surface, X_B_ is retained therefore needs to be clarified. In addition, it should be clarified what external factors (operational, influent WW) influence X_B_ retention in AGS SBR operation.

The main objective of this study was to better understand the fate of X_B_ during AGS SBR operation and ultimately to get insights about the presence and role of flocs in AGS systems. Therefore, the focus was on evaluating (1) to what extent X_B_ is retained during anaerobic plug-flow feeding and then during aerobic fully mixed conditions, (2) to what extent is the retention of X_B_ affected by operational variables (upflow feeding velocity v_ww_, mixing time) and influent composition on X_B_ retention in AGS SBR operation, and ultimately (3) to provide insights about the presence and role of flocs in AGS systems. Therefore, several different retention tests under plug-flow and fully-mixed conditions were conducted. Different types of biomass or mimics of biomass (AGS fed with acetate/propionate, glass beads, AGS fed with municipal WW, activated sludge flocs) and the effect of different v_ww_ during plug-flow feeding and mixing time during fully-mixed conditions were tested. Real municipal WW particles were used as X_B_ source, and COD mass-balances were conducted to quantify the extent of X_B_ retention in the different experiments. In addition, magnetic resonance imaging (MRI) was used to identify the mechanisms of X_B_ retention during plug-flow feeding.

## 2 Materials and methods

### 2.1 Experimental approach

Both *plug-flow,* MRI and *fully-mixed* tests were conducted to evaluate the fate of X_B_ in AGS SBR operation (Table 1). Primary effluent WW of the WW treatment plant (WWTP) of Eawag (Dübendorf, Switzerland) was used as the source of X_B_ during all tests. Anaerobic or anoxic redox conditions were kept during all tests in order to minimize degradation of X_B_.

**Table 1:** Details of the experimental approach, questions addressed and experimental variables.

	Hydraulic condition	Specific question addressed	Independent variables
Plug-flow test	Plug-flow	- Extent of X_B_ retention during anaerobic plug-flow feeding? - Effect of sludge bed type, v_ww_ and wastewater composition on X_B_ retention? - X_B_ retention distribution over bed height?	- Filter-bed composition (activated sludge flocs, real AGS, large granules, glass beads, or no biomass) - Upflow velocity within the reactor (v_ww_ = 1.0–5.0 m h^−1^) - Fixed (13 cm) or variable sludge bed height (0–20 cm)
MRI	Plug-flow	- X_B_ retention during plug-flow feeding: attachment or sedimentation in interstitial void space?	
Fully-mixed test	Fully-mixed	- X_B_ retention during aerobic fully-mixed conditions? - Effect of mixing time on X_B_ retention? - Does X_B_ attach to flocs, granules or both?	- Biomass type (increasing fractions of activated sludge flocs (0–100%) and large granules (100-0%) in 25% increments) - Mixing time (0.5–180 min)

### 2.2 Experimental set-up

#### 2.2.1 Plug-flow tests

**Fig. 2:**
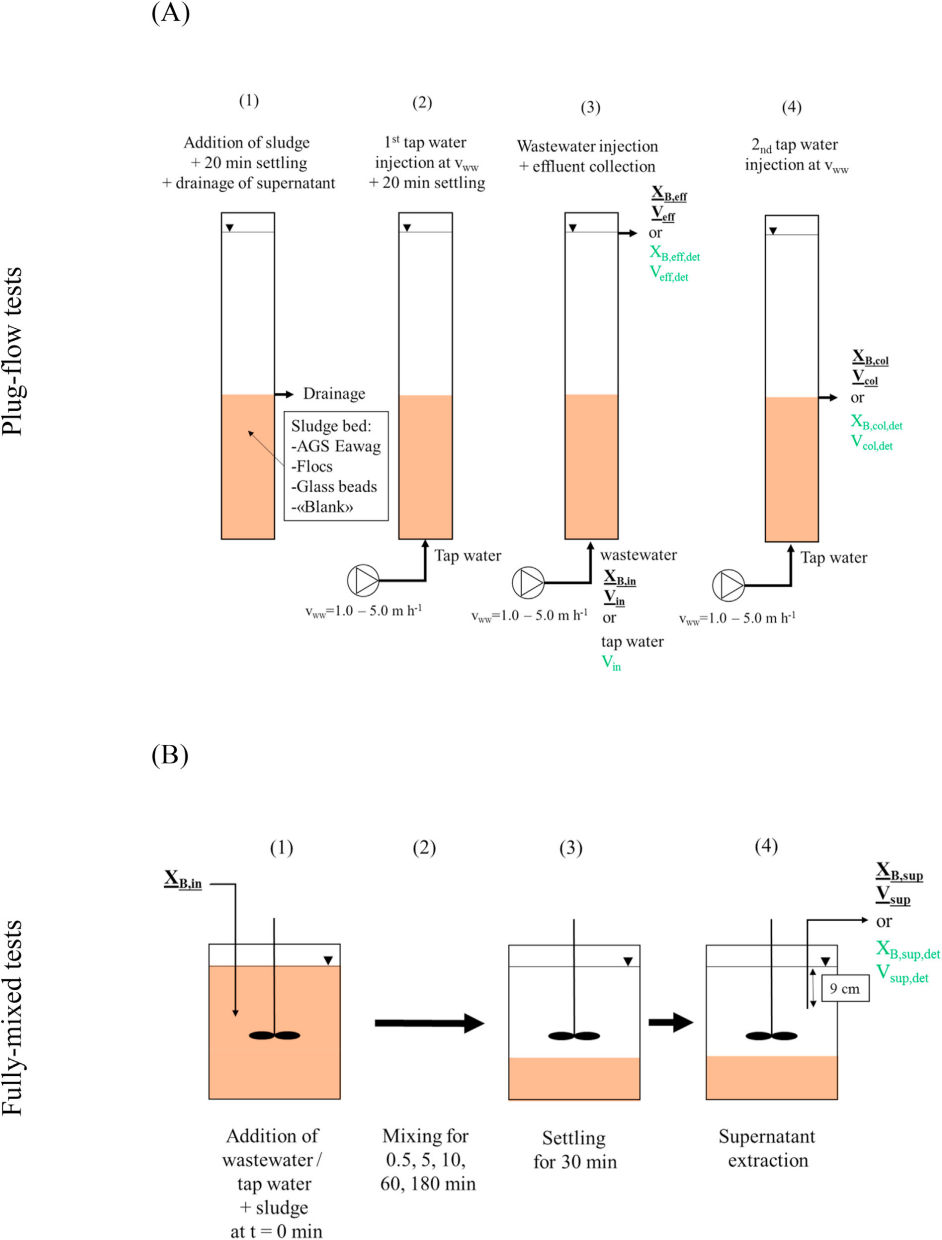
Schematic of procedure and sampling points during plug-flow tests (A) and fully-mixed tests (B). Underlined and bold measurement points indicate tests with WW addition, green measurement points indicate blank-tests without WW addition. (For interpretation of the references to colour in this figure legend, the reader is referred to the Web version of this article.)

*Plug-flow tests* were conducted to quantify X_B_ retention during anaerobic plug-flow feeding (Fig. 2A). Tests with different sludge beds of similar height (13 cm) were first conducted: empty bed (no biomass), activated sludge flocs, AGS fed with municipal WW (named “AGS Eawag”), and glass beads (2 mm) (see images of the different sludge in Supplementary Information Figs. S1A and C). Different v_ww_ (1.0–5.0 m h^−1^) and X_B_ influent concentration (variable) were tested (Table 1). Tests with variable bed height (0–20 cm) were then conducted to better understand the distribution of X_B_ retention over the sludge bed height (Table 1). Low and medium v_ww_ (1, 2.5 m h^−1^) and filter-bed composed of glass beads (d = 2 mm) were tested. In parallel to the tests with real WW X_B_ as influent, *blank plug-flow tests* were conducted (tap water injection instead of real WW) to account for X_B_ loss from the filter-bed during feeding. Columns with 2.5 and 5 cm inner diameter (working volume of 393 and 1963 mL, height of 82 and 100 cm, respectively) were used. The volume-exchange ratio (VER) was 1.3 during the plug-flow tests and the sludge volume after 30 min of settling (SV_30_) was 130 mL L^−1^. Very high VER >1.0 was used to make sure that some influent WW would exit the column through the effluent. The procedure of the *plug-flow tests* was as follows:

Step 1: Addition of sludge to the column to a targeted bed height of 13 cm (fixed sludge bed height tests) or variable from 0.5 to 20 cm (variable sludge bed height tests) after 20 min of settling. A settling duration of 20 min was sufficient to ensure a complete settling of the sludge during all tests. Supernatant removal above settled sludge bed using drainage ports.
Step 2: 1^st^ tap water injection from the bottom of the column using a peristaltic pump to refill the column (Heidolph, Germany). Second settling phase (20 min). Tap water was injected to refill the reactor, in order to mimic simultaneous fill-draw mode (constant volume operation), typically applied in full-scale AGS systems during feeding.
Step 3: Injection of 500 or 2500 mL (for small and large column, respectively)
  a. WW from the bottom of the reactor (normal *plug-flow tests*) with different v_ww_ (1.0–5.0 m h^−1^), effluent collection.
  b. Tap water from the bottom of the reactor (*blank plug-flow tests*) with different v_ww_ (1.0–5.0 m h^−1^), effluent collection.
Step 4: 2^nd^ tap water injection, drainage and collection of column supernatant.

#### 2.2.2 Fully-mixed tests

*Fully-mixed tests* were conducted to analyse X_B_ retention under fully-mixed conditions, representative of the aerobic phase of AGS systems (schematic Fig. 2B). The approach was based on Modin et al. (2015) and Jimenez et al. (2005). The influence of sludge composition, mixing time and influent X_B_ concentration was evaluated (Table 1). The sludge was composed of different ratios of large granules (>1 mm) 0–100% and flocs 100-0% in increments of 25% (see images of the different sludge in Supplementary Information Figs. S1B and D). Mixing times of 0.5, 5, 10, 60, 180 min and variable influent X_B_ concentrations were evaluated. All *fully-mixed tests* were conducted for a defined sludge composition. *Blank fully-mixed tests* were in addition conducted (tap water instead of real WW) to account for X_B_ loss from biomass. The procedure of the fully-mixed tests was as follow:

Step 1: Addition of 300 mL of biomass to 1 L glass beakers, with a target total suspended solids (TSS) concentration of 4 gTSS L^−1^.
  a. Addition of 700 mL of WW (normal *fully-mixed tests*)
  b. Addition of 700 mL of tap water (*blank fully-mixed tests*)
Step 2: Mixing for 0.5, 5, 10, 60 or 180 min. The mixing velocity gradient (G) was set to 3.3 s^−1^ using an apparatus with propellers, similar to the G-values maintained during aeration in the long-term lab-scale experiments performed at Eawag (Layer et al., 2019; Supplementary information S2).
Step 3: Settling for 30 min in order to separate biomass and supernatant.
Step 4: Collection of 50 mL of supernatant 9 cm underneath the water surface.

### 2.3 Analytical methods

TSS was quantified using standard methods (Apha, 2005). Sludge was separated using sieves of 0.25 mm (to separate flocs < 0.25 mm from granules > 0.25 mm) or 1 mm (to separate large granules > 1 mm from small granules, flocs and debris). Sieving of the different sludge fractions was performed by gently pouring the sludge into the sieve, and then washing the sieve with additional tap water. The particles retained by the sieve were collected by back-washing the cake that formed on the sieve with tap water. Size fractions were then quantified using TSS measurements. Total and soluble COD was measured using cuvette tests (LCK 114, 314, Hach-Lange, Germany, Kits). X_B_ was defined as the difference between total and soluble COD, measured after filtration at 0.45 μm using membrane filters (Macherey Nagel, Nanocolor Chromafil membranefilter GF/PET 0.45 μm, Germany). Samples were collected in 50 mL vials and homogenized for 1 min at 10′000 rpm (Ultra-Turrax, Ika, Germany) prior to total COD measurement. In our study, X_B_ refers to all COD fractions larger than 0.45 μm, including biodegradable and unbiodegradable fractions of particulate COD and possibly a fraction of the colloidal COD (Levine et al., 1985).

### 2.4 Calculations

COD mass-balances were performed to calculate X_B_ retention (%) during *plug-flow tests* (Eqs. (1), (2))) and *fully-mixed tests* (Eqs. (3), (4))). The mass-balance of *plug-flow tests* takes into account mass of X_B_ from influent, effluent, supernatant and is corrected for the mass of X_B_ that is detached during the tests (from *blank plug-flow tests*), Eqs. (1), (2)), Fig. 2A.

(1) $$f_{X_{B,PF,retained}}=\frac{M_{X_{B,injected}}-M_{X_{B,non-retained}}+M_{X_{B,detached}}}{M_{X_{B,injected}}}\times 100.\left[\%\right]$$

(2) $$f_{X_{B,PF,retained}}=\frac{X_{B,in}\cdot V_{in}-X_{B,eff}\cdot V_{eff}-X_{B,col}\cdot V_{col}+X_{B,eff,det}\cdot V_{eff,det}+X_{B,col,det}\cdot V_{col,det}}{X_{B,in}\cdot V_{in}}\cdot 100\cdot \left[\%\right]$$

where X_B,in_ is the X_B_ influent concentration and V_in_ is the injected volume into the column, X_B,eff_ is the X_B_ effluent concentration, V_eff_ the effluent volume, X_B,col_ is the X_B_ concentration in the column supernatant, V_col_ the volume of the column supernatant, X_B,eff,det_ is the detached X_B_ concentration in the effluent during *blank plug-flow tests*, V_eff,det_ the effluent volume during *blank plug-flow tests*, X_B,col,det_ is the detached X_B_ concentration of the column supernatant during *blank plug-flow tests* and V_col,det_ the volume of the column supernatant during *blank plug-flow tests*.

The *fully-mixed tests* mass-balance takes into account the mass of X_B_ which was added via WW, supernatant after a certain mixing time and is corrected for detaching mass of X_B_ (from *blank fully-mixed tests*), Eqs. (3), (4)), see Fig. 2B.

(3) $$f_{X_{B,mix,retained}}=\frac{M_{X_{B,injected}}-M_{X_{B,non-retained}}+M_{X_{B,detached}}}{M_{X_{B,injected}}}\cdot 100\cdot \left[\%\right]$$

(4) $$f_{X_{B,mix,retained}}=\frac{X_{B,in}\cdot V_{in}-X_{B,sup}\cdot V_{sup}+X_{B,sup,det}\cdot V_{sup,det}}{X_{B,in}\cdot V_{in}}\cdot 100\cdot \left[\%\right]$$

where X_B,in_ is the X_B_ concentration of the primary effluent WW added and V_in_ is the volume of the primary effluent WW added to the beaker at t = 0 min (0.7 L), X_B,sup_ is the X_B_ concentration of the supernatant after mixing for a given time and additional 30 min of settling, V_sup_ the total supernatant volume (1 L), X_B,sup,det_ is the X_B_ supernatant concentration during *blank fully-mixed tests*, and V_sup,det_ the supernatant volume during the *blank fully-mixed tests* (1 L).

The Reynolds number was calculated according to Eq. (5).

(5) $$Re=\frac{v\cdot d}{\nu }$$

where v is the upflow feeding velocity (m s^−1^), d the characteristic length (granule or glass-bead diameter during plug flow and magnetic resonance imaging tests) and ν the kinematic viscosity of water (1.003E-06 m^2^ s^−1^ at 20 °C).

### 2.5 Statistical analysis

Multivariate linear regression analysis was performed to identify the contribution of variance of independent variables on the variance of X_B_ retention (f_,XB,PF,retained_ and f_,XB,mix,retained_ were the target variables) during fixed bed height *plug-flow tests* (Section 3.1.1) and *fully-mixed tests* (Section 3.2). All data (independent and target variables) comprising *plug-flow tests* or *fully-mixed tests* were combined. The analysis was performed using ANOVA (Kaufmann and Schering, 2014) implemented in R (Version 3.6.0, R-Core-Team, 2018).

### 2.6 Magnetic resonance imaging (MRI)

MRI was used to differentiate between particles, granules, and void space during plug-flow feeding of a settled granular bed. MRI characterisations were carried out on a 200 MHz nuclear magnetic resonance spectrometer (Bruker Avance 200 SWB, Bruker BioSpin GmbH, Germany). The container (15.4 mL) was filled with fresh granules (d ≥ 1 mm, sieved) cultivated in SBR fed by acetate/propionate. Granules were collected after approx. 1 year of steady operation (Layer et al., 2019), and granular biomass was characterised by granules d > 1 mm resembling over 95% of biomass (TSS based). A low v_ww_ of 0.39 m h^−1^ was set during MRI tests to avoid channel formation, which is much lower than typically applied v_ww_ of 2 m h^−1^ in AGS operation (Derlon et al., 2016). The X_B_ source during MRI tests was sieved (d_p_ = 28–100 μm) municipal raw WW with TSS of 4.7 g L^−1^, collected at Eawag (Dübendorf, Switzerland), concentrated by centrifugation (3500 rpm, 10 min). A high concentration of TSS was necessary to ensure good separation of particles and granules based on intensity by MRI. A 1^st^ and 2^nd^ feeding were conducted in order to get an intermediary and final image of X_B_ retention during plug-flow feeding. 24 and 9 mL of influent WW were fed during the 1^st^ and 2^nd^ feeding, respectively.

Data analysis was performed using Matlab R2018b (MathWorks, USA) and Avizo 9.4 (Thermo Fisher Scientific, USA). The granular sludge bed was visualised with the *T*_*1*_-weighted images (see Supplementary Information Fig. S2, upper row). According to the signal intensity, particles appear the brightest, followed by granules and water filled void space. No signal (black) is obtained from exterior solid materials. For a clear differentiation between granules and particles based on signal intensity, predominantly *T*_*2*_-weighted images were conducted (see Supplementary Information Fig. S2, lower row), as the signal intensities of granules and particles were in a similar intensity range. A threshold value 6300 out of 2^15^ intensity values was chosen for predominantly *T*_*1*_-weighted images to separate granules and particles from void space and exterior parts. For predominantly *T*_*2*_-weighted images a threshold value 5000 was chosen to separate particles and exterior parts from granules and void space. The combination of both binary images allowed for a clear determination and quantification of the fractions. For a more detailed description of the applied method, please see Ranzinger et al. (2020).

## 3 Results

### 3.1 Retention of X_B_ during the anaerobic plug-flow feeding of AGS systems

#### 3.1.1 How is X_B_ retention influenced by influent WW composition, v_ww_ and biomass type in plug-flow conditions?

**Fig. 3:**
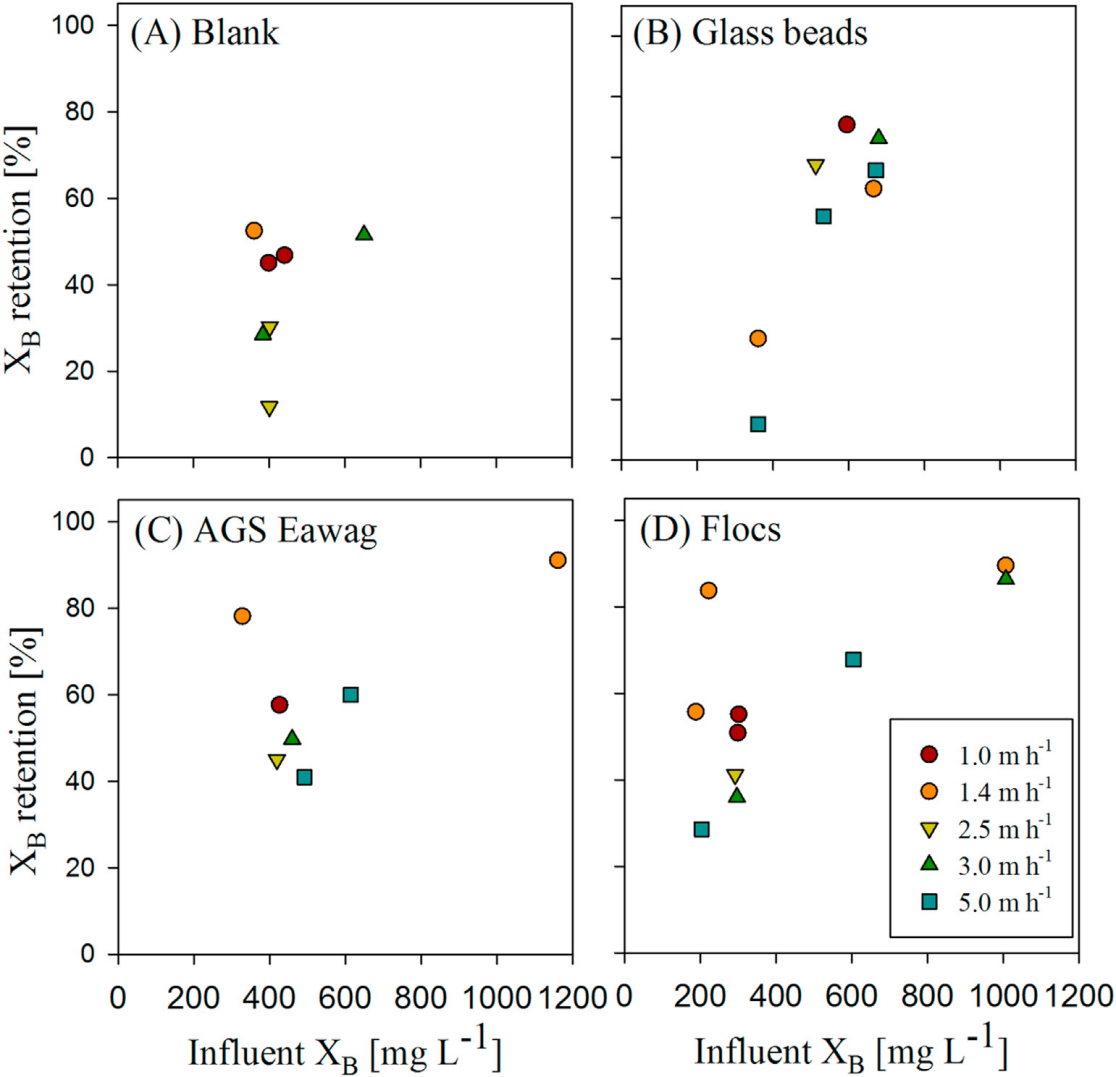
Retention of X_B_ in percent COD during plug-flow feeding for different influent X_B_ concentrations and different v_ww_ (1.0–5.0 m h^−1^) with different biomass compositions: A) Blank (no sludge bed), B) Glass beads (diameter 2 mm), C) AGS Eawag and D) Flocs.

X_B_ retention was evaluated during *plug-flow tests* (Fig. 3). X_B_ retention during plug-flow conditions varied between 10 and 90%. The concentration of X_B_ in the influent WW had major impact on X_B_ retention. Biomass composition and applied v_ww_ influenced X_B_ retention to a lesser extent.

Increasing X_B_ concentrations significantly increased X_B_ retention (p = 2.48E-07), independent of biomass composition or applied v_ww_. Specifically, high X_B_ influent concentrations > 600 mg L^−1^ resulted in X_B_ retention > 60%. Biomass composition also affected X_B_ retention during plug-flow conditions (p = 1.28E-03). In absence of a filter bed (blank test) 10–52% of influent X_B_ were retained (Fig. 3A). In presence of a filter bed, overall X_B_ retention is increased to > 60% on average (Fig. 3B–D). In addition, lower v_ww_ in general resulted in higher X_B_ retention (p = 0.022).

#### 3.1.2 How is X_B_ distributed over the bed height during plug-flow conditions?

**Fig. 4:**
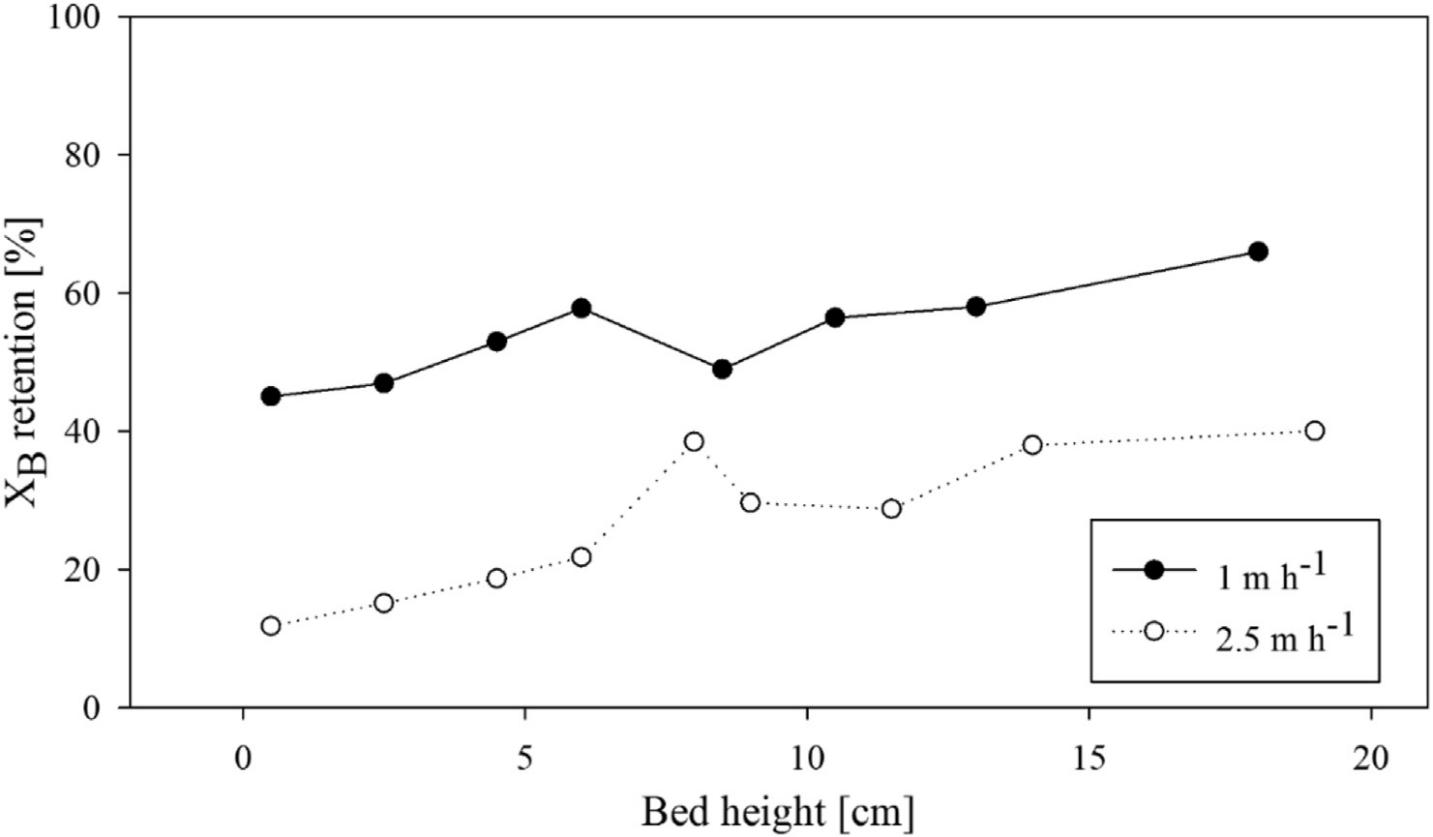
X_B_ retention in percent COD during plug-flow feeding at different locations through a sludge bed composed of glass bead (2 mm) at v_ww_ = 1 and 2.5 m h^−1^. Primary effluent WW was composed of X_B_ = 292 mg L^−1^ (v_ww_ = 1 m h^−1^) and X_B_ = 201 mg L^−1^ (v_ww_ = 2.5 m h^−1^).

A main question is where does the retention of X_B_ occur within the settled bed of AGS during plug-flow feeding? Results from the *plug-flow tests* with variable sludge-bed heights indicated that a gradient of X_B_ retention over the bed height existed (Fig. 4). Hereby, large amounts of X_B_ were retained at the bottom of the settled sludge bed. The larger was the upflow feeding velocity during the plug-flow feeding, the deeper was the penetration of X_B_ and hence the lower was the gradient of X_B_ retention within the settled sludge bed. Low v_ww_ of 1 m h^−1^ led to increased X_B_ retention at the bottom of the sludge bed. Almost 70% of final X_B_ retention occurred within the first 0.5 cm. On the other hand, higher v_ww_ of 2.5 m h^−1^ during feeding increased the penetration depth of X_B_, thus resulting in a more homogeneous distribution of X_B_ within the bed. The first 0.5 cm of the settled sludge bed retained 30% of the final retention in this case. Overall higher X_B_ retention at v_ww_ = 1 m h^−1^ were likely the result of a higher influent X_B_ concentration compared to the run at v_ww_ = 2.5 m h^−1^, which were 292 and 201 mg L^−1^ for v_ww_ = 1 and 2.5 m h^−1^, respectively. The Reynolds numbers were 0.6 and 1.4 for v_ww_ of 1 and 2.5 m h^−1^, respectively.

#### 3.1.3 Does X_B_ attach to granules surface or accumulate within interstitial voids of the sludge bed during plug-flow conditions?

**Fig. 5:**
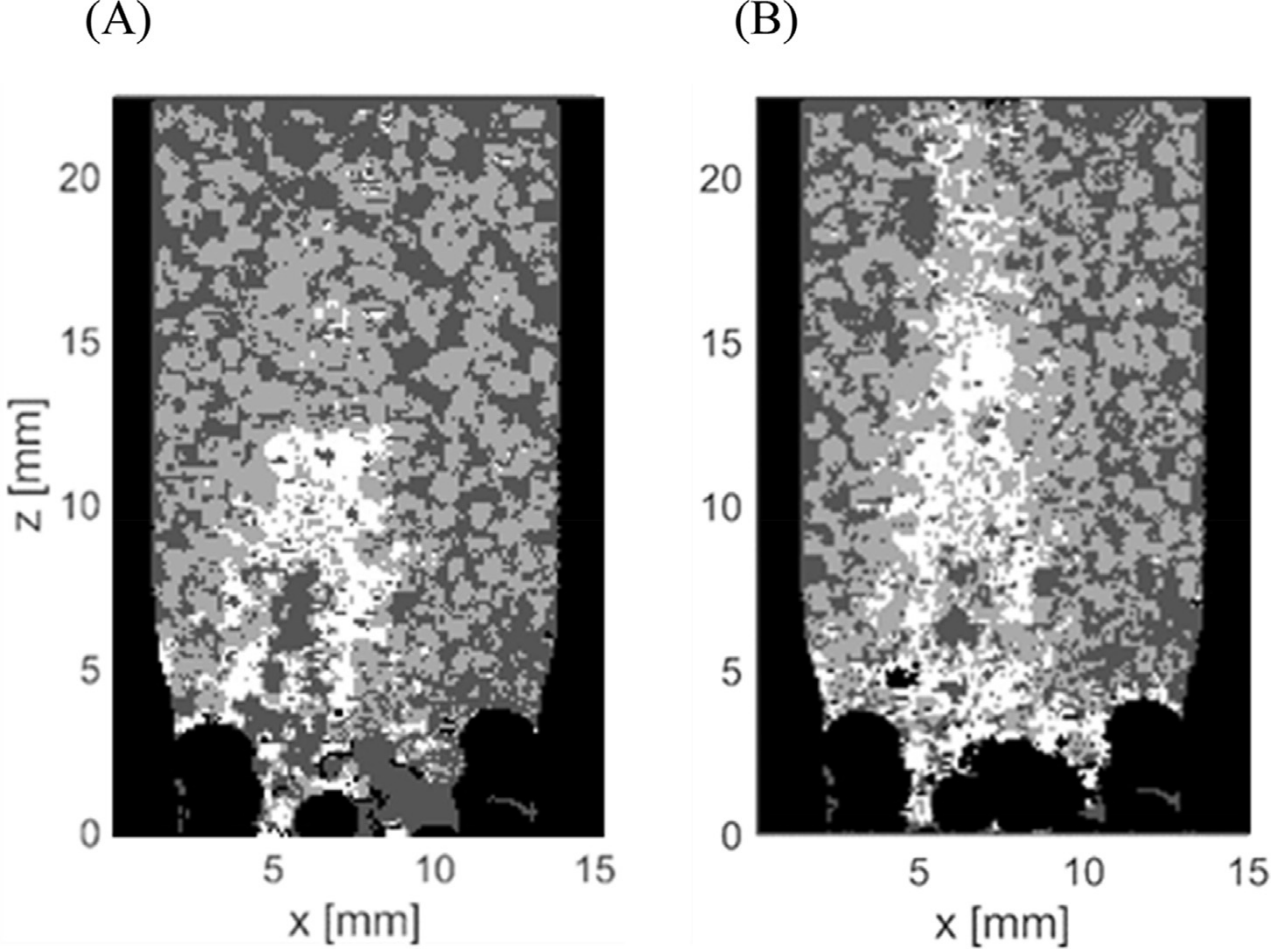
Quantified images after first (A) and second WW feeding (B). Quantified images are 2D sections out of the 3D measurements. X_B_ particles (white), granules (grey), water filled void space (dark grey) and exterior parts (black) can be differentiated.

**Fig. 6:**
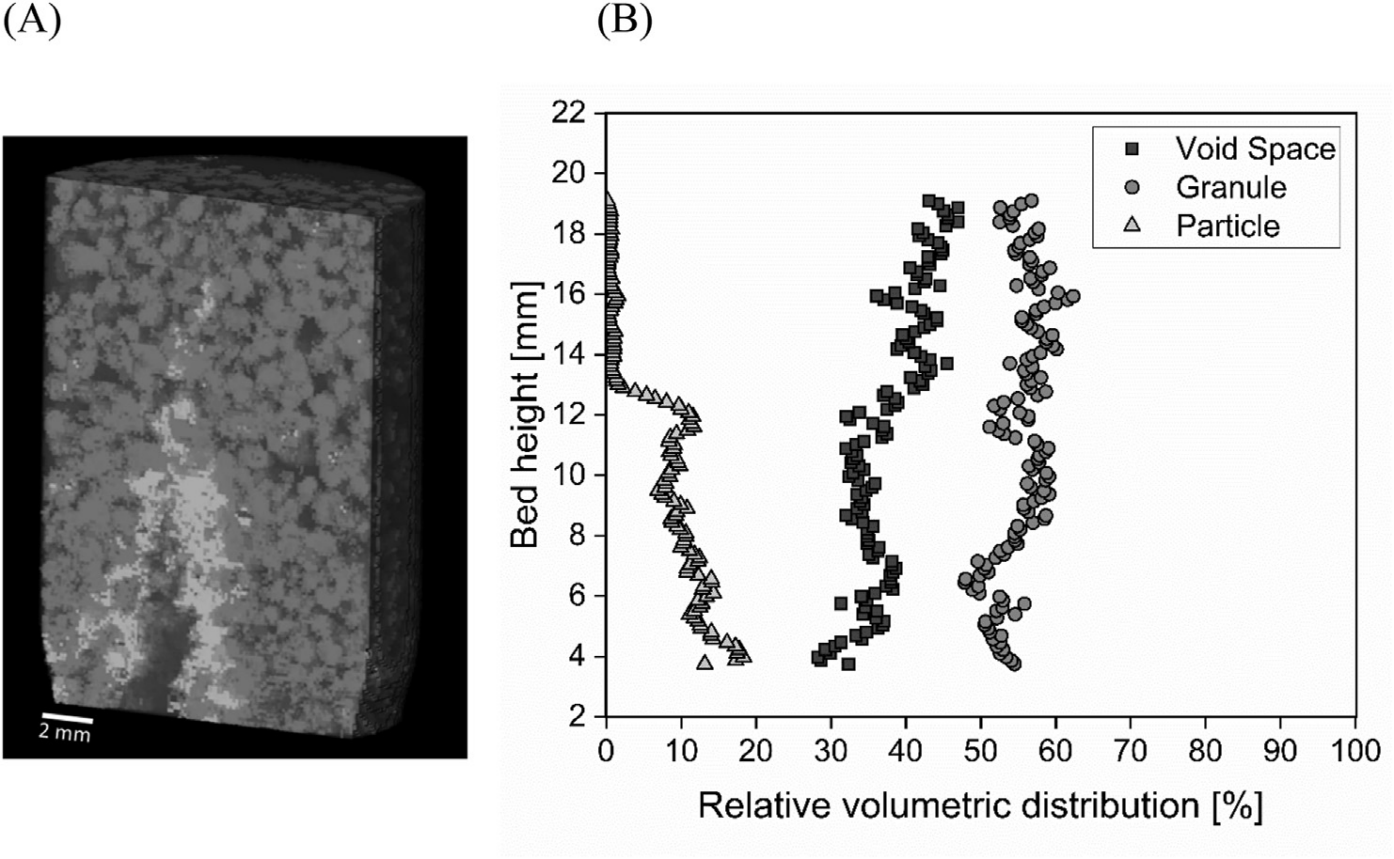
(A) 3D image of X_B_ particles inside the aerobic granular sludge bed after the 1^st^ feeding. WW particles (white), granules (grey), water filled void space (dark grey) and exterior parts (black) can be differentiated. (B) Relative volumetric distribution along the bed height after the 1^st^ feeding, calculated from the 3D image.

Our results from *plug-flow tests* helped to quantify the extent of X_B_ retention during plug-flow feeding and its spatial distribution over the height of the sludge bed. A major aspect is however to better understand if X_B_ is attached to the settled biomass after feeding, or if it simply accumulated within the bed without much contact. MRI tests were thus conducted to evaluate the spatial distribution of X_B_ within the settled granular sludge bed during anaerobic plug-flow feeding. Results from MRI tests demonstrated that X_B_ accumulated within the interstitial voids in the first few cm of the settled sludge bed, and that X_B_ accumulation was actually affected by both sedimentation and surface filtration (Fig. 5, Fig. 6).

Most X_B_ accumulated within the first 13 mm in vertical direction after the 1^st^ feeding (Fig. 5A, white colour, Fig. 6AB). Granules were pushed by the applied flow, creating channels and resulting in void space (Fig. 5A). Moreover, X_B_ hardly distributed horizontally within the granule bed. Instead, X_B_ was located mostly in the bottom of the chamber and additionally occupied the void space in vertical direction extending the inlet (Fig. 5A). Only minimal distribution of X_B_ in the x- and y-direction occurred despite the rather narrow chamber of the MRI, and no wall-effects were visible. After the 2^nd^ feeding X_B_ occupied even more of the void space and was distributed along the whole height of the chamber (Fig. 5B). Occupation of the void space by X_B_ was indicated by large white-coloured areas/volumes surrounding the preferential flow channel, created by the inlet flow in the centre of the column after the 1st and 2nd feeding (Figs. 5 and 6A). The Reynolds number during MRI tests was 0.1 assuming a granule diameter of d = 1.0 mm.

### 3.2 How is X_B_ retained during fully-mixed conditions?

**Fig. 7:**
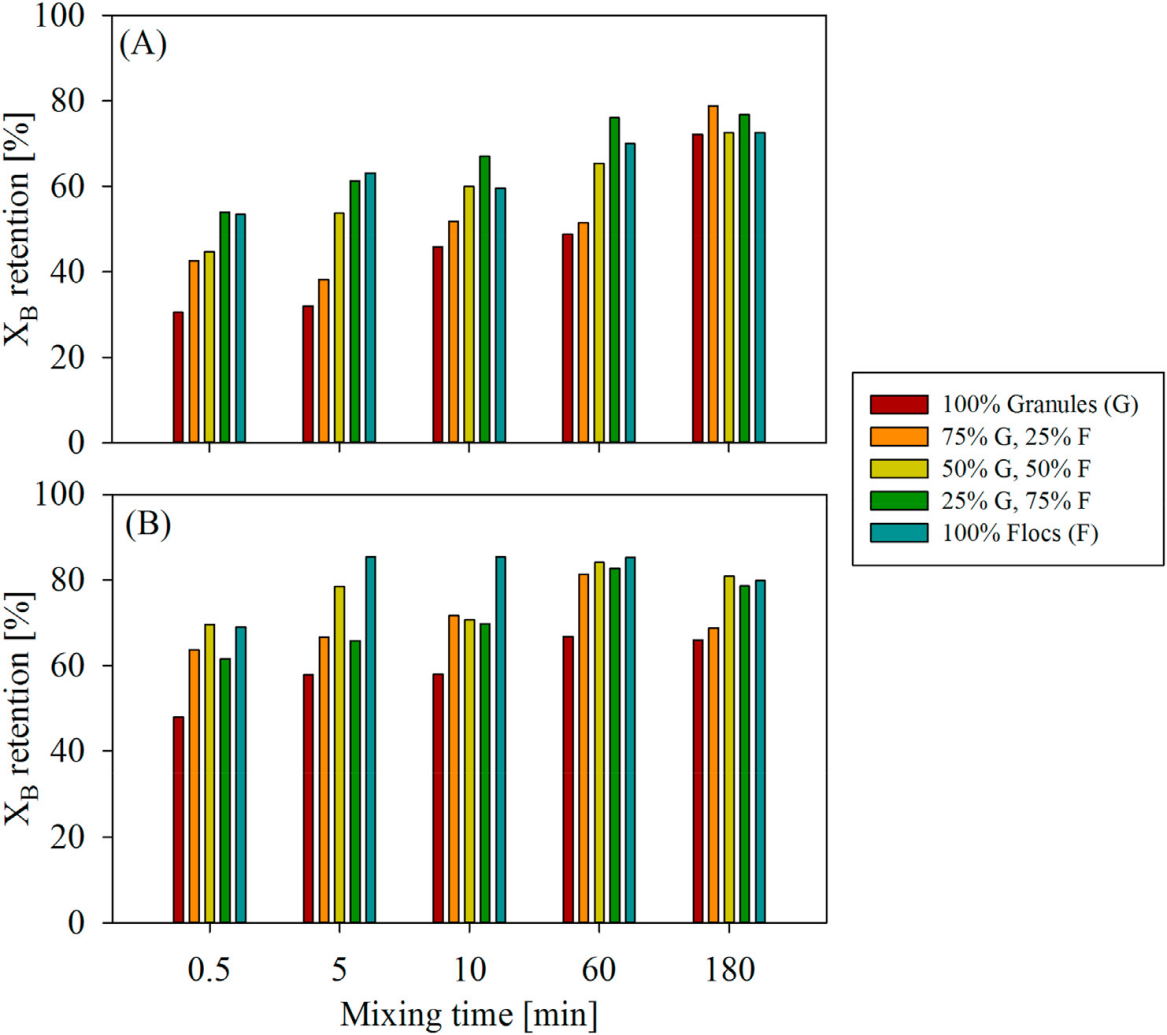
X_B_ retention in percent COD during fully mixed conditions displaying effects of different granule/flocs fractions for two different runs (A and B). Mixing times varied from 0.5 to 180 min. Primary effluent was used as X_B_ source (X_B_ concentration = 94 and 196 mgCOD L^−1^ for runs A and B, respectively).

If large fractions of influent X_B_ are retained within the settled sludge bed during anaerobic plug-flow feeding but not binding to the granules, it is then likely that X_B_ re-suspends and becomes available for attachment in aerobic fully-mixed conditions for both flocs and granules in AGS systems. Fully-mixed tests were thus conducted to better understand where X_B_ does attach during mixed conditions, *i.e.*, to granules or flocs (Fig. 7). Results from the *fully-mixed tests* indicated that an increasing floc fraction in the AGS significantly increased X_B_ retention during fully-mixed conditions in AGS systems (p < 1.0E-05), specifically in the first 60 min of mixing. Additionally, longer mixing times as well as higher influent X_B_ concentrations significantly increased X_B_ retention in AGS systems (all p < 1.0E-05).

Over 50% of the final X_B_ removal was achieved during the first 30 s of mixing if flocs were present in the biomass (Fig. 7). Reduced X_B_ retention was observed after 30 s in absence of flocs (>20% less X_B_ retention by 100% Granules, Fig. 7AB). However, the longer the mixing time was, the smaller were the differences in overall X_B_ retention between the different biomass compositions. After 3 h of mixing X_B_ retention was 60–85% among all biomass compositions and floc fractions were less important towards overall X_B_ retention (p = 0.14) (Fig. 7AB). It must be noted that the total biomass concentration during fully-mixed tests was held constant, independent of different granule-flocs fractions, which further highlighted the impact of flocs on X_B_ retention in mixes of granules and flocs. Influent X_B_ concentration also contributed to the overall level of X_B_ retention. The increased X_B_ influent concentration of *fully-mixed test* B (196 mgCOD L^−1^, Fig. 7B) led to overall higher X_B_ retention, independent of mixing time or biomass composition, when compared to the lower X_B_ influent concentration of fully-mixed test A (94 mgCOD L^−1^, Fig. 7A).

## 4 Discussion

### 4.1 X_B_ accumulates within the sludge bed during plug-flow feeding but does not attach to granules

Our first main result is that X_B_ accumulated predominantly within the voids at the bottom of the settled sludge bed, thus indicating that X_B_ retention was governed by sedimentation and surface filtration during plug-flow feeding (Fig. 3, Fig. 4, Fig. 5, Fig. 6). If X_B_ retention was governed by sedimentation and surface filtration, it is then likely that only a minor fraction of X_B_ is actually in contact with the granules during anaerobic plug-flow feeding (Fig. 5, Fig. 6; Ranzinger et al., 2020).

We propose that X_B_ retention through sedimentation and surface filtration during plug-flow feeding of AGS systems is a 3-step process, consisting of (1) channel formation, (2) settling of X_B_ and (3) surface filtration. Firstly, influent flow causes slight redistribution of granules, which locally enlarges void space and then forms channels in upward direction within the settled sludge bed. Secondly, influent X_B_ settles within the channels. The channels are progressively filled up by the settling of X_B_, ultimately resulting in a filter-cake. Thirdly, influent X_B_ is then strained by the filter-cake and surface filtration occurs (Maroudas and Eisenklam, 1965). With continuing influent WW injection, the filter-cake consisting of X_B_ is being pushed upwards. Attachment of X_B_ to the granules during plug-flow feeding is thus limited. However, our results do not allow us to conclude about actual contact between filter-cake and the granule surface, since the resolution of MRI is too coarse (Ranzinger et al., 2020). A minor fraction of X_B_ could thus be in contact to the granules during anaerobic plug-flow feeding.

Our results also indicated that the influent X_B_ concentration and upflow velocity determined the extent of X_B_ retention during plug-flow feeding. The upflow feeding velocities applied at pilot-scale but also in full-scale AGS system are ranging from 0.5 to 5 m h^−1^, with a typical value of 2 m h^−1^ when treating municipal WW (Derlon et al., 2016; Wagner et al., 2015a; Pronk et al., 2015b). The settling velocity of influent X_B_ particles in the size range of 45–200 μm in diameter is 0.8–16 m h^−1^ (specific gravity 1.2 kg L^−1^) or 4.0–75 m h^−1^ (specific gravity 2.0 kg L^−1^) (Stokes, 1851; Levine et al., 1985; Johnson et al., 1996). The settling velocities of X_B_ particles are in general larger than the values of upflow feeding velocities. However, the upflow velocity of the influent WW must be corrected for the porosity of the settled sludge bed, with a typical value of 0.52 (Van Dijk et al., 2020). The actual upflow velocity within the sludge bed pores therefore increases by a factor of 1.9, to values of 1.0–9.6 m h^−1^. In general, the actual upflow velocities are in the same range as the settling velocities of influent X_B_ particles. However, large X_B_ particles are strongly affected by sedimentation and could therefore play an important role in the initial formation of a filter-cake at the bottom of the settled sludge bed. Smaller X_B_ particles that are transported through advection could then be retained through surface filtration by the filter-cake (Maroudas and Eisenklam, 1965). Higher X_B_ concentrations usually coincide with larger particle diameters (Sophonsiri and Morgenroth, 2004). Influent WW composed of high X_B_ concentrations will thus lead to increased settling of X_B_ at the bottom of the sludge bed and fast formation of a filter-cake during the anaerobic plug-flow feeding. The overall particle size distribution entering the AGS SBR is determined by whether primary treatment via primary clarification or a similar filtration or sedimentation step is implemented or not (Levine et al., 1991). Colloids (particles d < 1 μm) are prone to diffusion, and can indeed diffuse into the granules located at the bottom of the sludge bed during plug-flow feeding (Ranzinger et al., 2020). Retention of colloidal particles is therefore governed by inherently different mechanisms compared to retention of X_B_ particles, which cannot diffuse into the biofilm (Polson, 1950). It must be noted that MRI tests were conducted using a very high X_B_ loading, in order to increase the image quality. Therefore results gained from MRI (Fig. 5, Fig. 6) likely overemphasised the magnitude but not the occurrence of sedimentation and surface filtration as X_B_ retention mechanisms during plug-flow feeding.

We therefore propose that a large fraction of X_B_ is retained at the bottom of the settled sludge bed through sedimentation and surface filtration and is thus not attached to biomass. Combining limited attachment to biomass and slow hydrolysis in anaerobic plug-flow feeding conditions suggests that large fractions of X_B_ are not hydrolysed during anaerobic plug-flow feeding conditions (Henze and Mladenovski, 1991). Large fractions of influent X_B_ could therefore re-suspend once aerobic fully-mixed aerobic conditions are applied (Ranzinger et al., 2020), in analogy to particle re-suspension during backwash of granular media filters (Amirtharajah, 1985).

### 4.2 Large fractions of X_B_ are retained by flocs during fully-mixed conditions

Another main finding of our study is that X_B_ re-suspends during fully-mixed conditions, *e.g.*, once aeration starts, and is available for attachment onto both granules and flocs. A main question is whether X_B_ will then attach preferentially to the flocs or to the granules.

During the first 60 min of mixing, the presence of flocs increased X_B_ retention by more than 20%, in comparison to the “100% granules” case (Fig. 7). We hypothesize that X_B_ retention is mostly achieved by flocs through rapid attachment, due to the very large specific surface area of flocs (flocs TSS fraction 20%, flocs-to-granules surface area ratio 939-to-1, Supplementary Information S4, Andreadakis, 1993; Mihciokur and Oguz, 2016; Jimenez et al., 2005). Granules, on the other hand, have a much smaller specific surface area and are much lower in number (Supplementary Information S4). In addition, the surface of mature granules is often rather smooth when flocs are also present in the AGS, and granules do not offer many locations for attachment in comparison to odd-shaped, ramified flocs. Reduced X_B_ removal and decreased X_B_ removal rates by biofilm systems is linked to limited active adsorption sites (Boltz and La Motta, 2007). We thus propose that flocs have a competitive advantage over granules to retain X_B_ through attachment during fully-mixed aerobic conditions, due to their physical structure despite their minor fraction in AGS systems treating municipal WW (10–30% TSS-based; Layer et al., 2019). If X_B_ is attaching rapidly and preferentially to the flocs, only little X_B_ is then left for attachment onto the granules. Attachment of X_B_ onto the granules was much slower compared to X_B_ attachment to mixtures of flocs and granules, or solely flocs (Fig. 7).

Previous studies indeed indicate that the contribution of biofilms to the retention and hydrolysis of X_B_ is quite limited during fully-mixed conditions. Particles > 1 μm are typically considered the most difficult to be removed in biofilm systems (Levine et al., 1991). In moving bed biofilm reactors (MBBR) used for the treatment of municipal wastewater, no reduction in TSS usually occurs in the MBBR stage (Åhl et al., 2006). In general, reduced hydrolysis of X_B_ has been reported for biofilm systems in comparison to conventional activated sludge systems (Janning et al., 1998; Morgenroth et al., 2002). Actually, several studies even suggested that hydrolysis in biofilm systems is carried out in the bulk phase rather than at the biofilm surface (Rohold and Harremoës, 1993; Larsen and Harremoës, 1994a, 1994b). Those findings suggest that the contribution of biofilms to X_B_ hydrolysis is rather small, due to the limited attachment of X_B_ onto biofilms. AGS systems treating municipal WW are now often regarded to as hybrid biofilm systems (Layer et al., 2019). Therefore, we hypothesize that in hybrid systems such as AGS, flocs outcompete granules in X_B_ retention through attachment once mixing is applied.

### 4.3 Practical implications

Attachment of X_B_ was quite limited during anaerobic plug-flow conditions, and full retention of X_B_ was then achieved in aerobic fully-mixed conditions. Retention of X_B_ during *fully-mixed tests* were performed using very high X_B_-to-biomass ratios (70/30 v/v), and final X_B_ retention was > 80% in all tests. Thus, complete removal of X_B_ can be expected during the aerobic fully-mixed phase in full-scale AGS SBR operation. Flocs retained a large fraction of X_B_ through rapid attachment after mixing was applied. Therefore, it is very likely that (1) X_B_ will be fully hydrolysed within the SBR cycle (Henze et al., 2000) and that (2) the majority of hydrolysis products are consumed within the floc micro-environment, too (Martins et al., 2011). Flocs will thus always co-exist with granules in AGS systems as long as the WW contains organic substrate in the form of X_B_. Aggressive wash-out of flocs via short settling times still is a common start-up and operational strategy in AGS SBR operation (Adav et al., 2008). We however propose that too aggressive wash-out of flocs is neither desirable nor expedient in AGS systems treating municipal WW, even at the cost of decreased settling performance (Layer et al., 2019). It is likely that too high wash-out of flocs in AGS systems treating X_B_-rich municipal WW leads to increased X_B_ attachment, hydrolysis and utilisation by the granules. An increased aerobic utilisation of X_B_ by the granules would then result. Aerobic utilisation of organic substrate by the granules was linked to filamentous outgrowth, loss of nutrient removal performance and/or granule breakage and process failure, eventually (Sturm et al., 2004; De Kreuk et al., 2010; Derlon et al., 2016; Haaksman et al., 2020). To date, it is still under debate if flocs have other important functions in AGS systems like, *e.g.*, if their contribution towards nutrient removal is significant or negligible, and whether their presence is desirable or not (Ali et al., 2019; Layer et al., 2019, 2020). Therefore, more research is required on the specific function of flocs in AGS systems treating municipal WW.

X_B_ retention can be optimised by *e.g.* introducing an anaerobic-mixed phase after plug-flow feeding (Layer et al., 2019). An increased attachment of X_B_ to flocs and granules during anaerobic conditions would be the result. However, prior research has indicated that anaerobic hydrolysis of X_B_ originating from municipal WW can be limited (Jabari et al., 2016). Thus, anaerobic X_B_ degradation by introducing anaerobic-mixing could be limited. Another option could aim at minimising X_B_ in the influent to the AGS stage through advanced pre-treatment such as micro-sieving or chemically enhanced pre-treatment (Sancho et al., 2019). Pre-fermentation of captured X_B_ in primary treatment could indeed enhance AGS performance in low-strength municipal WW conditions (Yuan et al., 2020; Vollertsen et al., 2006). However, more research is required to identify feasible operational strategies and technologies to improve X_B_ retention, degradation and utilisation in AGS-based WWTP.

Reynolds numbers calculated for *plug-flow tests* indicated laminar flow conditions during plug-flow feeding at lab-scale. It must be noted that turbulent flow conditions could occur during the feeding phase of a full-scale AGS SBR, depending on the design of the influent WW distribution system, *e.g.,* due to scarce injection nozzle distribution. In such case, two distinct zones might exist, where the first zone (*e.g.,* bottom 10–50 cm of the settled sludge bed) experiences turbulent flow conditions and could act as a fluidized bed. Within the fluidized bed attachment of X_B_ to biomass could be possible. The second zone above the fluidized bed would experience laminar flow conditions, where similar X_B_ retention mechanisms as observed in our study likely occur. However, to date no detailed information on full-scale AGS SBR injection hydraulics are available, and thus, considerations are highly speculative.

## 5 Conclusions

1. During anaerobic plug-flow feeding of AGS SBR, X_B_ is retained within the interstitial voids of the settled sludge bed, but with minimal attachment. In the subsequent fully-mixed phase X_B_ then attaches preferentially to the flocs.
2. X_B_ retention results from the combined mechanisms of sedimentation and surface filtration that occur at the bottom of the settled sludge bed during anaerobic plug-flow feeding. Up to 70% of the final X_B_ retention occurred within the first 0.5 cm of the settled sludge bed. The attachment of X_B_ onto the granules is thus limited during anaerobic plug-flow feeding.
3. The extent of X_B_ retention during plug-flow feeding is determined by WW composition (influent X_B_ concentration), v_ww_ and sludge bed composition. High influent X_B_ concentrations and low v_ww_ increase X_B_ retention.
4. A large fraction of influent X_B_ likely re-suspends during aerobic fully-mixed conditions. Rapid X_B_ retention after 0.5–60 min of mixing occurs if flocs are present in the biomass. Therefore, X_B_ attaches preferentially to flocs and only a small fraction of X_B_ attaches to granules.
5. Flocs are an important biomass fraction in AGS systems treating municipal WW rich in X_B_. Too high wash-out of flocs is not desirable in those conditions.

## Declaration of competing interest

The authors declare that they have no known competing financial interests or personal relationships that could have appeared to influence the work reported in this paper.

## References

[bib1] Adav S.S., Lee D.-J., Show K.-Y., Tay J.-H. (2008). Aerobic granular sludge: recent advances. Biotechnol. Adv..

[bib2] Åhl R.M., Leiknes T., Ødegaard H. (2006). Tracking particle size distributions in a moving bed biofilm membrane reactor for treatment of municipal wastewater. Water Sci. Technol..

[bib3] Ali M., Wang Z., Salam K.W., Hari A.R., Pronk M., Van Loosdrecht M.C.M., Saikaly P.E. (2019). Importance of species sorting and immigration on the bacterial assembly of different-sized aggregates in a full-scale Aerobic granular sludge plant. Environmental Science & Technology.

[bib4] Amirtharajah A. (1985). The interface between filtration and backwashing. Water Res..

[bib5] Andreadakis A.D. (1993). Physical and chemical properties of activated sludge floc. Water Res..

[bib6] Apha (2005). Standard Methods for the Examination of Water and Wastewater.

[bib7] Boltz J.P., La Motta E.J. (2007). Kinetics of particulate organic matter removal as a response to bioflocculation in aerobic biofilm reactors. Water Environ. Res..

[bib8] De Kreuk M.K., Kishida N., Tsuneda S., Van Loosdrecht M.C.M. (2010). Behavior of polymeric substrates in an aerobic granular sludge system. Water Res..

[bib9] Derlon N., Wagner J., Da Costa R.H.R., Morgenroth E. (2016). Formation of aerobic granules for the treatment of real and low-strength municipal wastewater using a sequencing batch reactor operated at constant volume. Water Res..

[bib10] Guimarães L.B., Wagner J., Akaboci T.R.V., Daudt G.C., Nielsen P.H., Van Loosdrecht M.C.M., Weissbrodt D.G., Da Costa R.H.R. (2018). Elucidating performance failures in use of granular sludge for nutrient removal from domestic wastewater in a warm coastal climate region. Environ. Technol..

[bib11] Haaksman V.A., Mirghorayshi M., Van Loosdrecht M.C.M., Pronk M. (2020). Impact of aerobic availability of readily biodegradable Cod on morphological stability of aerobic granular sludge. Water Res..

[bib12] Henze M., Gujer W., Mino T., Van Loosdrecht M.C.M. (2000). Activated Sludge Models Asm 1, Asm 2, Asm2d and Asm 3.

[bib13] Henze M., Mladenovski C. (1991). Hydrolysis of particulate substrate by activated sludge under aerobic, anoxic and anaerobic conditions. Water Res..

[bib14] Jabari P., Yuan Q., Oleszkiewicz J.A. (2016). Potential of hydrolysis of particulate Cod in extended anaerobic conditions to enhance biological phosphorous removal. Biotechnol. Bioeng..

[bib15] Janning K.F., Le Tallec X., Haffemoës P. (1998). Hydrolysis of organic wastewater particles in laboratory scale and pilot scale biofilm reactors under anoxic and aerobic conditions. Water Sci. Technol..

[bib16] Jimenez J.A., La Motta E.J., Parker D.S. (2005). Kinetics of removal of particulate chemical oxygen demand in the activated-sludge process. Water Environ. Res..

[bib17] Johnson C.P., Li X., Logan B.E. (1996). Settling velocities of fractal aggregates. Environmental Science & Technology.

[bib18] Kaufmann J., Schering A. (2014). Analysis of Variance Anova.

[bib19] Larsen T.A., Harremoës P. (1994). Degradation mechanisms of colloidal organic matter in biofilm reactors. Water Res..

[bib20] Larsen T.A., Harremoës P. (1994). Modelling of experiments with colloidal organic matter in biofilm reactors. Water Sci. Technol..

[bib21] Layer M., Adler A., Reynaert E., Hernandez A., Pagni M., Morgenroth E., Holliger C., Derlon N. (2019). Organic substrate diffusibility governs microbial community composition, nutrient removal performance and kinetics of granulation of aerobic granular sludge. Water Res..

[bib22] Layer M., Garcia Villodres M., Hernandez A., Reynaert E., Morgenroth E., Derlon N. (2020). Limited simultaneous nitrification-denitrification (Snd) in aerobic granular sludge systems treating municipal wastewater: Mechanisms and practical implications. Water Res..

[bib23] Levine A.D., Tchobanoglous G., Asano T. (1985). Characterization of the size distribution of contaminants in wastewater: Treatment and reuse implications. Water Pollution Control Federation.

[bib24] Levine A.D., Tchobanoglous G., Asano T. (1991). Size distributions of particulate contaminants in wastewater and their impact on treatability. Water Res..

[bib25] Maroudas A., Eisenklam P. (1965). Clarification of suspensions: a study of particle deposition in granular media: Part I—some observations on particle deposition. Chem. Eng. Sci..

[bib26] Martins A.M.P., Karahan Ö., Van Loosdrecht M.C.M. (2011). Effect of polymeric substrate on sludge settleability. Water Res..

[bib27] Metcalf, Eddy, Abu-Orf M., Tchobanoglous G., Stensel H.D., Tsuchihashi R., Burton F., Bowden G., Pfrang W. (2014). Wastewater Engineering: Treatment and Resource Recovery, Fifth Edition, Revised by.

[bib28] Mihciokur H., Oguz M. (2016). Removal of oxytetracycline and determining its biosorption properties on aerobic granular sludge. Environ. Toxicol. Pharmacol..

[bib29] Modin O., Saheb Alam S., Persson F., Wilén B.-M. (2015). Sorption and release of organics by primary, anaerobic, and aerobic activated sludge mixed with raw municipal wastewater. PloS One.

[bib30] Morgenroth E., Kommedal R., Harremoës P. (2002). Processes and modeling of hydrolysis of particulate organic matter in aerobic wastewater treatment – a review. Water Sci. Technol..

[bib31] Polson A. (1950). The some aspects of diffusion in solution and a definition of a colloidal particle. J. Phys. Colloid Chem..

[bib32] Pronk M., Abbas B., Al-Zuhairy S.H.K., Kraan R., Kleerebezem R., Van Loosdrecht M.C.M. (2015). Effect and behaviour of different substrates in relation to the formation of aerobic granular sludge. Appl. Microbiol. Biotechnol..

[bib33] Pronk M., De Kreuk M.K., De Bruin B., Kamminga P., Kleerebezem R., Van Loosdrecht M.C.M. (2015). Full scale performance of the aerobic granular sludge process for sewage treatment. Water Res..

[bib34] R-Core-Team (2018). R: A Language and Environment for Statistical Computing. http://www.R-project.org.

[bib35] Ranzinger F., Matern M., Layer M., Guthausen G., Wagner M., Derlon N., Horn H. (2020). Transport and retention of artificial and real wastewater particles inside a bed of settled aerobic granular sludge assessed applying magnetic resonance imaging. Water Res..

[bib36] Rocktäschel T., Klarmann C., Ochoa J., Boisson P., Sørensen K., Horn H. (2015). Influence of the granulation grade on the concentration of suspended solids in the effluent of a pilot scale sequencing batch reactor operated with aerobic granular sludge. Separ. Purif. Technol..

[bib37] Rohold L., Harremoës P. (1993). Degradation of non-diffusible organic matter in biofilm reactors. Water Res..

[bib38] Sancho I., Lopez-Palau S., Arespacochaga N., Cortina J.L. (2019). New concepts on carbon redirection in wastewater treatment plants: a review. Sci. Total Environ..

[bib39] Sophonsiri C., Morgenroth E. (2004). Chemical composition associated with different particle size fractions in municipal, industrial, and agricultural wastewaters. Chemosphere.

[bib40] Stokes G.G. (1851). On the Effect of the Internal Friction of Fluids on the Motion of Pendulums.

[bib41] Sturm B., Irvine R., Wilderer P. (2004). The effect of intermittent feeding on aerobic granule structure. Water Sci. Technol..

[bib42] Van Dijk E., Pronk M., Van Loosdrecht M. (2020). A settling model for full-scale aerobic granular sludge. Water Res..

[bib43] Van Dijk E.J.H., Pronk M., Van Loosdrecht M.C.M. (2018). Controlling effluent suspended solids in the aerobic granular sludge process. Water Res..

[bib44] Vollertsen J., Petersen G., Borregaard V.R. (2006). Hydrolysis and fermentation of activated sludge to enhance biological phosphorus removal. Water Sci. Technol..

[bib45] Wagner J., Guimarães L.B., Akaboci T.R.V., Costa R.H.R. (2015). Aerobic granular sludge technology and nitrogen removal for domestic wastewater treatment. Water Sci. Technol..

[bib46] Wagner J., Weissbrodt D.G., Manguin V., Da Costa R.H.R., Morgenroth E., Derlon N. (2015). Effect of particulate organic substrate on aerobic granulation and operating conditions of sequencing batch reactors. Water Res..

[bib47] Yuan Q., Gong H., Xi H., Wang K. (2020). Aerobic granular sludge formation based on substrate availability: effects of flow pattern and fermentation pretreatment. Front. Environ. Sci. Eng..

