# Supplementary material for: Particulate substrate retention in plug-flow and fully-mixed conditions during operation of aerobic granular sludge systems
Source: Water Res X. 2020 Oct 28;9:100075. doi: 10.1016/j.wroa.2020.100075 (PMC7645637; doi:10.1016/j.wroa.2020.100075)
Supplement: Multimedia component 1 [file mmc1.pdf]

Particulate substrate retention in plug-flow and fully-mixed conditions during operation of aerobic granular sludge systems

SUPPLEMENTARY INFORMATION

M. Layer[*,**], K. Bock[*], F. Ranzinger[***], H. Horn[***], E. Morgenroth[*,**], N. Derlon[*]

* Eawag, Swiss Federal Institute of Aquatic Science and Technology, Überlandstrasse 133, 8600 Dübendorf, Switzerland

** ETH Zürich, Institute of Environmental Engineering, 8093 Zürich, Switzerland

*** Engler-Bunte-Institute, Karlsruhe Institute of Technology, 76131 Karlsruhe, Germany

Email of the first author: manuel.layer@eawag.ch

Corresponding author: Nicolas Derlon (nicolas.derlon@eawag.ch)

S1 Sludge images

(A)

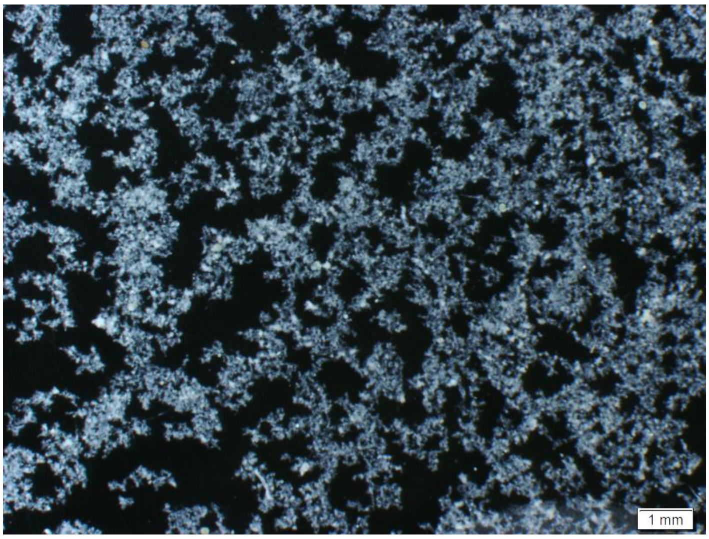

(B)

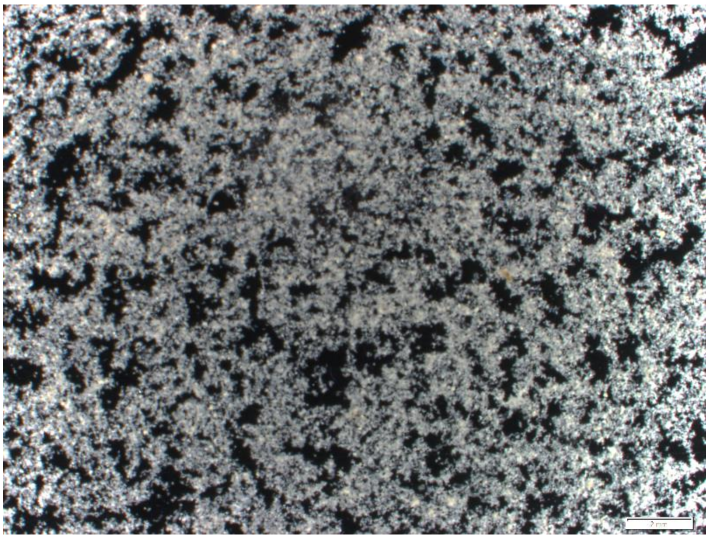

(C)

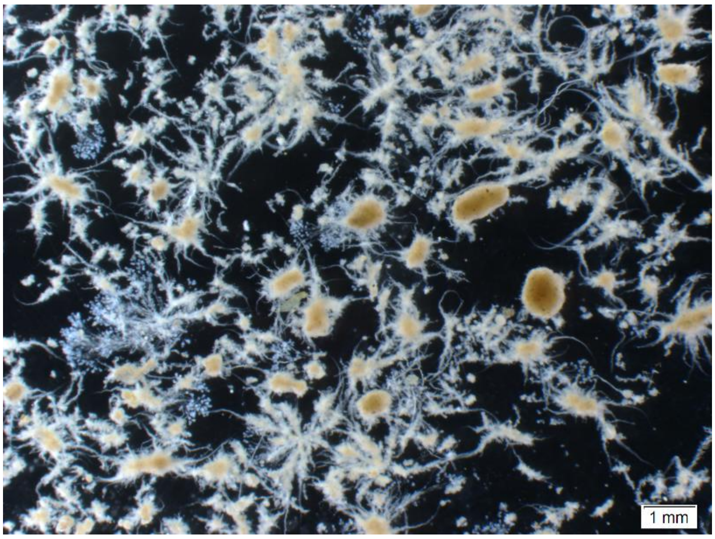

(D)

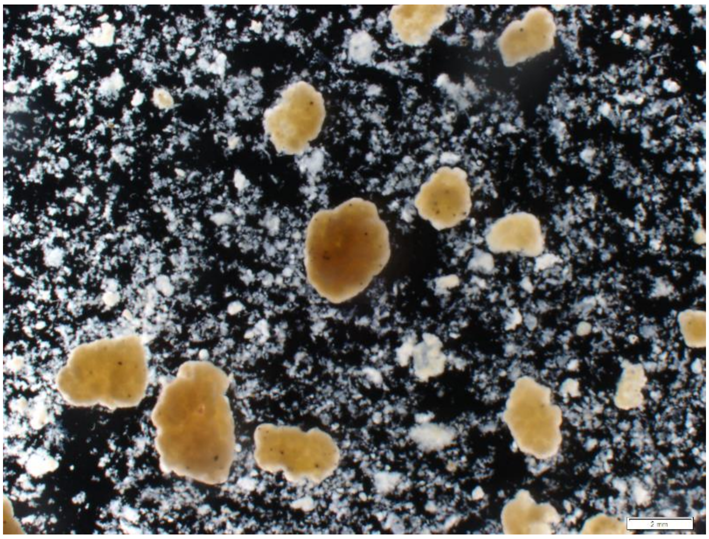

Figure S1: Sludge images during plug-flow (A,C) and fully-mixed tests (B,D). (A) Activated sludge flocs of WWTP Neugut, Dübendorf, Switzerland during plug-flow tests (size bar = 1.0 mm), (B) activated sludge flocs of WWTP Neugut, Dübendorf, Switzerland used during fully-mixed tests (size bar = 2.0 mm), (C) AGS Eawag used during plug-flow tests (size bar = 1 mm), (D) granules cultivated on acetate + propionate as sole carbon source used during fully-mixed tests (size bar = 1.0 mm) prior to sieving at 1.0 mm during long-term experiments (Layer et al., 2019).

S2 Calculation of velocity gradient (G) for fully-mixed tests

G was calculated using Eqs. S1, S2 and S3.

$$G = \sqrt{\frac{\varepsilon}{\mu}} \qquad \text{(Eq. S1)}$$

$$\varepsilon = \frac{P_0 \cdot N^3 \cdot D^5}{V} \qquad \text{(Eq. S2)}$$

where ε is the dissipated power per volume (Nm s^{-1} m^{-3}), μ the dynamic viscosity of water (0.001 kg s^{-1} m^{-1}), P_0 the power number (-), N the impeller speed (rpm), D the impeller diameter (m) and V the beaker volume (m^3).

The power number has been estimated by the Reynolds number (Eq. S3)

$$Re = \frac{\rho \cdot N^3 \cdot D^5}{V} \qquad \text{(Eq. S3)}$$

Where ρ is the density of water (998 kg m-3), N the impeller speed (rpm), D the impeller diameter (m) and ν the kinematic viscosity of water (0.01 N s m^{-2}).

Two different apparatus were used during jar tests, which used different propellers, see Table S1. For turbulent conditions (Re > 10 000) during jar tests, P_0 has been estimated to 0.5 for small propellers and 0.7 for paddles (Leentvaar et al., 1978).

Table S1: Comparison of Apparatus 1 and 2 used during the «jar tests» and the corresponding parameters to calculate G. The impeller speed N of Apparatus 1 and 2 was adjusted to match the values of G for both devices.

	Apparatus 1	Apparatus 2
Propeller diameter [m]	0.07	0.075
Power number P_0 [-]	0.5	0.7
Impeller speed N [rpm]	140	112
G [s^{-1}]	3.3	3.3

S3 Image processing and analysis of 3D MRI images

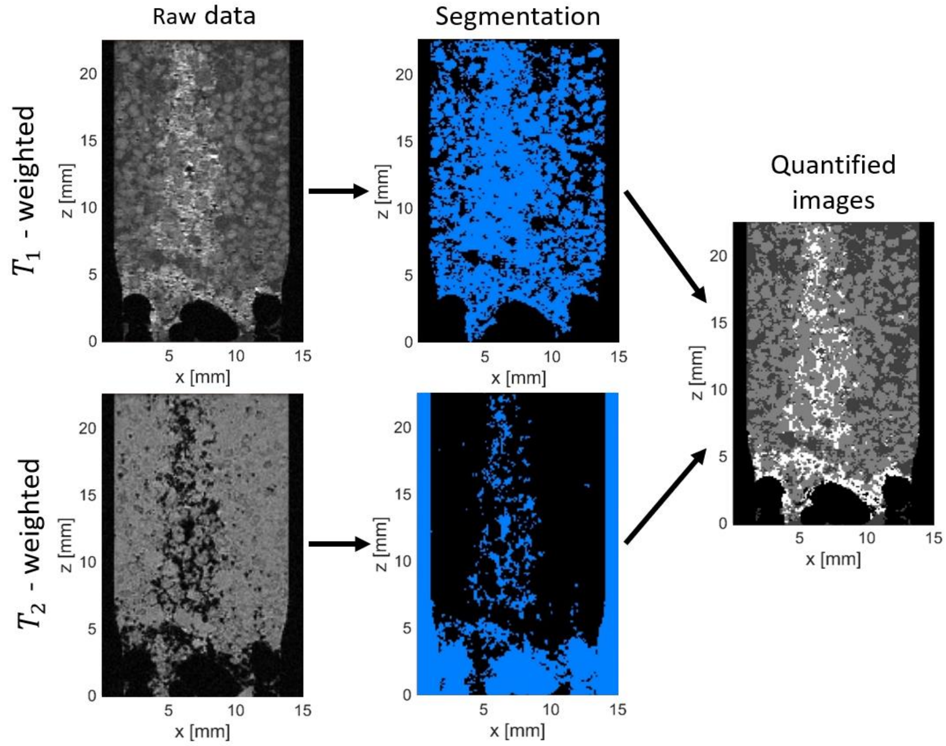

Figure S2: Image processing and analysis of 3D MRI images. Thresholds were manually set for predominantly T_1-weighted (upper row) and T_2-weighted images (lower row) to generate quantified images that allow differentiating between WW particles (white), granules (grey), void space (dark grey) and exterior parts (black).

S4 Flocs / granules number and surface area calculations

A total TSS of 6 gTSS L^{-1} is assumed of which 80% are granules, 20% are flocs.

For flocs, the number concentration (# L^{-1}) is calculated using a TSS of 1.2 gTSS L^{-1} (20% of 6 gTSS L^{-1}, assumption) a density of 1.02 g cm^{-3} (Andreadakis, 1993), a mean size of 40 µm (Andreadakis, 1993), resulting in a square geometric area of 1.6E-05 cm^2, an assumed thickness of 10 µm, resulting in an individual floc volume of 1.6E-08 cm^3. The number concentration then is calculated to 7.3E+07 # L^{-1}. The specific surface area of activated sludge flocs is 150 m^2 $gTSS^{-1}$ (Andreadakis, 1993).

For granules, the number concentration is calculated using a TSS of 4.8 gTSS L^{-1} (80% of 6 gTSS L^{-1}, assumption), a density of 1.08 g cm^{-3} (Winkler, 2012), a mean granules size of 1 mm

(assumption), resulting in a spherical individual granule volume of 5.24E-04 cm^3. The number concentration then is calculated to 8.5E+03 # L^{-1}. The specific surface area of granules (assuming a smooth surface) is calculated to 5.6E-03 m^2 $gTSS^{-1}$.

The number ratio of flocs:granules is then calculated to 8624:1, and the surface area ratio of flocs:granules is calculated to 938:1.

References

ANDREADAKIS, A. D. 1993. Physical and chemical properties of activated sludge floc. *Water Research,* 27**,** 1707-1714.

LAYER, M., ADLER, A., REYNAERT, E., HERNANDEZ, A., PAGNI, M., MORGENROTH, E., HOLLIGER, C. & DERLON, N. 2019. Organic substrate diffusibility governs microbial community composition, nutrient removal performance and kinetics of granulation of aerobic granular sludge. *Water Research X***,** 100033.

LEENTVAAR, J., WERUMEUS BUNING, W. G. & KOPPERS, H. M. M. 1978. Physico-chemical treatment of municipal wastewater. Coagulation-flocculation. *Water Research,* 12**,** 35-40.

WINKLER, M. K. H. 2012. *Magic Granules.* PhD Thesis, TU Delft.
